# Characterization and Expression Profiling Analysis of Calmodulin Genes in Response to Salt and Osmotic Stresses in Pear (*Pyrus bretschneideri* Rehd.) and in Comparison with* Arabidopsis*

**DOI:** 10.1155/2017/7904162

**Published:** 2017-03-08

**Authors:** Jun Tang, Jing Lin, Xiaogang Li, Qingsong Yang, Qunkang Cheng, Zong-Ming (Max) Cheng, Youhong Chang

**Affiliations:** ^1^Jiangsu Key Laboratory for Horticultural Crop Genetic Improvement, Institute of Horticulture, Jiangsu Academy of Agricultural Sciences, Nanjing 210014, China; ^2^Department of Plant Sciences, University of Tennessee, Knoxville, TN 37996, USA; ^3^Department of Entomology and Plant Pathology, University of Tennessee, Knoxville, TN 37996, USA

## Abstract

A genome-wide identification and cloning of CaM genes in pear was conducted and in compared with* Arabidopsis* that indicated a conserved expansion of CaM genes in pear, and PbCaMs and AtCaMs had a similar distribution of cis-elements and expressions in response to salt and osmotic stress. In particular, PbCaM1 and PbCaM3 were both significantly upregulated in response to salt and osmotic stress in pear.

## 1. Introduction

Calcium (Ca^2+^) is an important second messenger in eukaryotic cells. Ca^2+^ signaling plays diverse and essential functions in many aspects of plant development and stress responses. Many abiotic factors, including salt, temperature, light, and osmotic stress, modulate Ca^2+^ signals that are then recognized and translated into downstream responses by Ca^2+^ sensors [1–4]. Ca^2+^ sensors can bind Ca^2+^ and induce a conformational change in the sensor that promotes an interaction with downstream effectors or modulates its own catalytic activity [5]. Most Ca^2+^ sensors utilize the elongation factor- (EF) hand motif, a helix-loop-helix structure, to bind Ca^2+^; with each EF-motif binding a single Ca^2+^ ion [6]. Several families of Ca^2+^ sensors have been identified in higher plants based on the presence of EF-hand motifs including calmodulin proteins (CaMs) and calmodulin-like proteins (CMLs), calcineurin B-like proteins (CBLs), and Ca^2+^-dependent protein kinases (CPKs) [4, 5, 7–12]. Due to their commonality, CaMs as a Ca^2+^ sensor in eukaryotes are well known. On the other hand, CMLs, CBLs, and CDPKs may be restricted to just plants [5, 6, 13]. CaMs lack effector modules such as the kinase domain in CDPK proteins and lack any other identifiable functional domain except the EF-hand motif. CaMs transduce Ca^2+^ signals through their interaction with a series of target proteins (CaM-binding proteins [CaMBP]), which control a multitude of cellular functions [5, 7, 14].

All of the typical plant CaM family members are highly similar to animal CaMs and contain four EF-hand motifs. CaMs are organized into two distinct globular domains connected by a long flexible helix, and each of the globular domains contains one pair of intimately linked EF-hands that serves as the basic functional unit [5]. Seven CaMs (*AtCaM1*–*AtCaM7*) have been identified in the* Arabidopsis* genome [5], that function as one of several intracellular transducers that transmit information from diverse developmental and environmental stimuli [15, 16]. Studies have demonstrated that CaMs play an important role in activating stress-related proteins and, thus by implication, play a role in augmenting abiotic stress tolerance in plants [3, 6, 16]. For instance, CaM can regulate the heat shock-induced expression of HSP90 and HSP70 in sorghum [17]. In* Arabidopsis*, CaM7 can physically interact with HY5 and regulate its expression to promote photomorphogenic growth [18]. AtCaM3 functions as a downstream factor of NO signal transduction, which is involved in heat shock signal transduction [19, 20]. Overexpression of CaM3 can decrease the level of COR (cold regulated) transcripts in transgenic* Arabidopsis* plants [21]. Moreover, CaMs reportedly can activate AtMPK8, which subsequently suppresses wound-induced ROS accumulation [15]. AtMYB2 was identified as a CaM-binding protein that regulates the response to salt and dehydration stress [14]. AtCaMBP25 (a CaM-binding protein) is targeted to the nucleus and acted as a negative effector of osmotic and salt stress responses [1]. Interestingly, recent study [14] indicated that CaMs can also interact with group IId proteins of the entire WRKY subfamily of transcription factors which contain conserved Ca^2+^-dependent CaM-binding domains in regulating abiotic stress adaptation [14, 22, 23] and those proteins contain conserved Ca^2+^-dependent CaM-binding domains [14]. In addition, some plant-specific CaM-binding proteins, such as At-BT1, AT-BT2, and At-BT5 are induced in AtBZIP60-overexpressed plants, conferring the salt tolerance, suggested these At-BT proteins may be involved in protecting plants from salt stress [10, 24]. These data show diverse functions of CaMs, CaM-binding proteins, and their synergistic effects to deal with multiple stresses, offering further encouragement to explore the function of CaM gene regulated abiotic stress responses in plants.

Due to the multiple functions of CaMs and the increase in the availability of sequenced plant genomes, many* CaM* genes have been identified at the whole genome level in several plant species including* Arabidopsis* [5, 7], rice [25], and several species in the Solanaceae family [26]. Pear (*Pyrus bretschneideri*), a species within Rosaceae, is cultivated worldwide as a fruit tree, whose growth, development, and productivity are frequently affected by abiotic stress, such as salinity and drought [27]. Although the pear genome has been released [28], an analysis of the* CaM* gene family at the whole genome level has not been conducted. Additionally, few studies on specific pear* CaM* genes have been published. The present study reported genome-wide analysis of the* CaM* gene family in pear. Analyses included an examination of gene structure, the presence of conserved motifs, phylogeny, chromosomal locations, synteny analysis, presence of cis-regulatory elements, and expression profiles in response to salt and osmotic stress. A comparison with the expression of* CaM* in* Arabidopsis* was also conducted. Results indicated a conserved evolution of the* CaM* gene family in pear and that some* PbCaM* genes were obviously upregulated in response to salt and osmotic stresses. These data provide new insight into the conserved evolution and expression of the pear* CaM* gene family and their potential role in the response of pear to abiotic stress.

## 2. Materials and Methods

### 2.1. Identification and Characterization of CaM Genes in Pear

The genome sequence of pear (*P. bretschneideri*) was obtained from the pear genome project website (https://peargenome.njau.edu.cn). The* Arabidopsis* CaM protein sequences [5, 7] were from The* Arabidopsis* Information Resource (TAIR; http://www.arabidopsis.org/). BLASTP and TBLASTN were used to identify putative CaM family proteins in the pear proteome and genome using* Arabidopsis* CaM proteins as queries with default parameters. Sequences with an identity >80% against the queries [7, 12] were collected and analyzed using Pfam (http://pfam.xfam.org/) [29] and SMART (http://smart.embl-heidelberg.de/) [30] to identify the proteins with four conserved EF-hand domains and without other known functional domains. Then, the remaining nonredundant sequences were as putative CaM candidates in pear. Simultaneously, to avoid false positives and erroneous assembly errors, the full-length cDNA sequence was cloned for each of the putative* PbCaM* genes using gene-specific primers (Table S1 in Supplementary Material available online at https://doi.org/10.1155/2017/7904162) based on cDNA templates obtained from pear (*P. bretschneideri* cv “Dangshansuli”) leaves. PCR products were cloned and sequenced to confirm the identity of each* PbCaM* gene. A total of four sequenced genes were collected as true* CaM* genes in pear and named according to their genomic locations (Table 1).

### 2.2. Sequence Alignments, Structure Analysis, and Phylogenetic Analyses

Multiple sequence alignments were carried out using CLUSTALW 2.0 software [31] and conserved sequences were viewed and edited using GeneDoc (http://genedoc.software.informer.com/2.7/). Phylogenetic trees that included CaM sequences from pear (*P. bretschneideri*),* Arabidopsis* (*A. thaliana*), rice (*Oryza sativa*), tomato (*Solanum lycopersicum*), tobacco (*Nicotiana benthamiana*), and potato (*Solanum tuberosum*) were generated using the maximum-likelihood (ML) method and bootstrap analysis with 1000 replicates in MEGA7 software [31, 32]. Data for CaM proteins from rice, tomato, tobacco, and potato were obtained from Zhao et al. [26]. Gene structures were analyzed through aligning their coding sequences and their corresponding genomic sequences with the online tool GSDS2.0 (http://gsds.cbi.pku.edu.cn/index.php) [33]. Secondary structure analysis of CaM proteins was performed using JPred 4 (http://www.compbio.dundee.ac.uk/jpred4/index.html).

### 2.3. Protein Properties, Conserved Motifs, and Cis-Element Analysis

The molecular weight (MW) and isoelectric point (pI) of CaM proteins were calculated using ProtParam (http://web.expasy.org/protparam/). The distribution of conserved motifs within the CaM proteins was determined using MEME Suite software (http://meme-suite.org/index.html) [34] with default settings. A 1000 bp upstream genomic sequence above the transcription start site (ATG) was obtained for all of the identified* CaM* genes from the pear and the* Arabidopsis* genome sequence. The obtained sequences were submitted to the PlantCARE database (http://bioinformatics.psb.ugent.be/webtools/plantcare/html/) [35]. Lastly, stress-related cis-elements were collected and shown using an in-house Perl script.

### 2.4. Chromosomal Location and Synteny Analysis

To map the corresponding* CaM* loci on pear and* Arabidopsis* chromosomes, the genome annotation files of pear and* Arabidopsis* were obtained from the pear genome project website (https://peargenome.njau.edu.cn) and TAIR (http://www.arabidopsis.org/), respectively. To detect synteny of the* CaM* genes in pear and* Arabidopsis*, the whole genome synteny block data within/between pear and* Arabidopsis* genomes were collected from the Plant Genome Duplication Database (http://chibba.agtec.uga.edu/duplication/) [36]. Chromosomal locations and synteny relationships for the* CaM* family genes within and between the pear and* Arabidopsis* genome were examined to show their duplications and synteny relationships using Circos [37].

### 2.5. Plant Material and Treatments

The newly growing shoots with young leaves were collected from the 20-old-year pear (*P. bretschneideri* “Dangshansuli”) in the national germplasm orchard of the Institute of Horticulture, Jiangsu Academy of Agricultural Sciences, Nanjing, China. The samples were treated according to methods previously described [38]. Briefly, the shoots with young leaves were firstly placed in hydroponic containers containing 1/2 MS [39] solution (pH 5.8) for pretreatment of 14 days at 22 ± 2°C, 16 h light/8 h dark photoperiod, and 60–70% humidity conditions in growth chambers. The robust shoots with young leaves were used for stress treatments, and then the shoots were exposed to a 1/2 MS solution (pH 5.8) containing 200 mM NaCl and 15% (w/v) polyethylene glycol (PEG6000), respectively. Control was exposed to the same growth conditions but were grown only in 1/2 MS solution (pH 5.8) without the addition of any salt or polyethylene glycol. Young leaves were sampled according to [40] after 0, 12, 24, and 48 h of each treatment, respectively. For each treatment, three biological replicates were included and all samples were flash frozen in liquid nitrogen and stored at −80°C until further analysis.

### 2.6. RNA Isolation, cDNA Synthesis, and Cloning of PbCaM Genes

Total RNA was isolated from treated leaves using the TaKaRa MiniBEST Plant RNA Extraction Kit (TaKaRa, Dalian, China; Code no. 9769) according to the manufacturer's instructions. RNA concentration and integrity was quantified using a Nanodrop1000 (Thermo Scientific, Wilmington, DE) and its integrity was checked by electrophoresis in a 1.5% (w/v) agarose gel. Total RNA (2 *μ*g) from pear leaves was reverse transcribed into cDNA by PrimeScript™ 1st Strand cDNA Synthesis Kit (TaKaRa, Dalian, China; Code number 6110A) in order to clone* PbCaM* genes. PCR was performed with PrimeSTAR® Max DNA Polymerase (TaKaRa, Code no. R045A), and amplification conditions were as follows: 95°C for 5 min, followed by 35 cycles of 95°C for 30 s, 55~58°C for 2 min, and 72°C for 30 s and a final extension at 72°C for 10 min. PCR products were purified and cloned into the pMD19-T vector (TaKaRa). Positive clones were sequenced in order to confirm the identity and sequence length of the cloned* PbCaM* genes.

### 2.7. Quantitative Real-Time RT-PCR (qRT-PCR) and Expression Profiling Analysis

A total of 2 *μ*g of total RNA was used to synthesize cDNA with the PrimeScript RT reagent Kit with gDNA Eraser (Perfect Real Time) Kit (Takara, Code no. RR047A) according to the manufacturer's instructions. The cDNA reaction mixture was diluted 1 : 10 with EASY Dilution for Real-Time PCR (Takara). SYBR® Premix Ex Taq™ II (Takara, Code no. RR820A) was used to analyze gene expression with a 7500 Fast Real-Time PCR System (Applied Biosystems, USA). qRT-PCR was carried out in 96-well, optical reaction plates. The PCR reaction was performed in a total volume of 20 *μ*L containing 0.5 *μ*M of each primer (1 *μ*L), 20 ng/*μ*L cDNA (1 *μ*L), and 1x SYBR Premix Ex Taq II (10 *μ*L) and additional ddH_2_O to make a final volume of 20 *μ*L. The gene-specific primers were designed according to nonconserved region sequences of each* PbCaM* gene using the program Beacon Designer 8.10 (http://www.premierbiosoft.com/), and then the hit primer pairs of each gene were subjected to realignment with the coding sequences of the whole pear genome using BLASTn. Finally, the primer pair was collected for qRT-PCR only when they both matched the same* PbCaM* gene (Table S1). Pear* Actin2/7* and* UBI* genes were used as internal standards for subsequent normalization of the data as previously described by Xu et al. [41]. The settings of qRT-PCR conditions and the calculation of relative gene expression are performed according to methods described previously [38]. All of the experiments were examined using three biological replicates and three technical replicates for all of the genes. The relative expression levels were normalized to a value of 1 in the respective 0 h treated samples. The data of each gene was shown using the mean ± standard deviation (SD).

In addition, the expressions of* CaM* genes in* Arabidopsis* were analyzed based on the AtGenExpress (http://jsp.weigelworld.org/AtGenExpress/resources/) [42] and the heat map was generated using Cluster 3.0 software [43].

## 3. Results and Discussion

### 3.1. Identification and Characterization of CaM Genes in Pear

**Figure 1:**
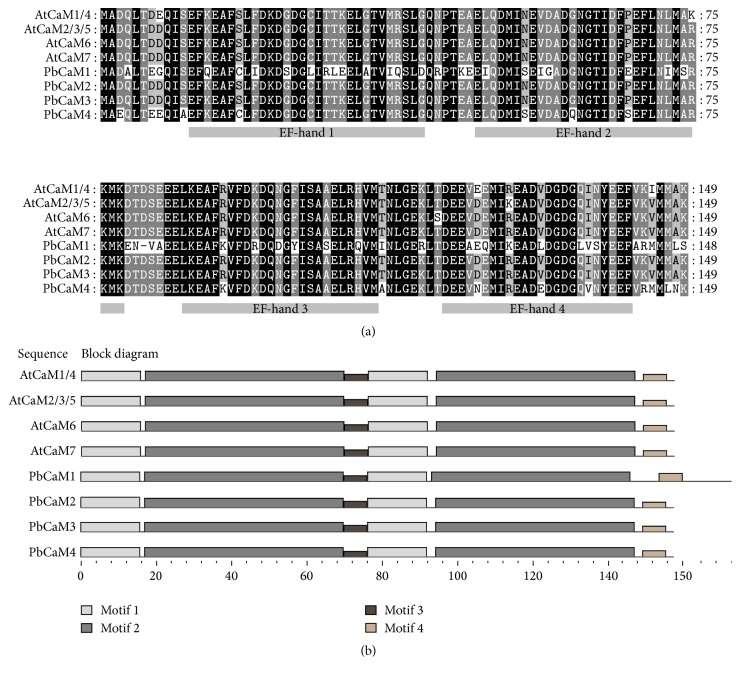
Alignment and conserved motifs of CaM proteins in pear (*P. bretschneideri*) and* A. thaliana*. (a) Multiple sequence alignment of PbCaM proteins were carried out using CLUSTALW 2.0 and a conserved motif analysis was performed using MEME (http://meme.nbcr.net/meme/). (b) The conserved protein motifs were detected using MEME Suite software (http://meme-suite.org/index.html) with default settings. The background dense represented the size of sequences similarity.

Through combination of the local BLASTP and TBLASTN searches using typical CaM protein sequences from* Arabidopsis* as queries, nonredundant hit sequences with >80% identity [7, 12] were collected and examined for the presence of conserved domains using Pfam [29] and SMART [30]. Sequences containing four EF-hand domains and having no other known functional domains were considered as putative* PbCaM* genes. Table 1 listed these four genes which displayed similar characteristics with all of the common CaM protein properties in* Arabidopsis* [4, 5, 7, 12]. These four putative* CaM* genes were cloned to confirm the reliability of genome-wide identification of* CaM* genes in pear. Sequencing of the cloned cDNA further showed complete consistency with those obtained from the genome-wide prediction. These four genes were named and submitted to the NCBI database under the accession numbers KU950328, KU950329, KU950330, KU950331, and KU950332 (Table 1). The length of the coding region of the four* PbCaM*s ranged from 450 to 564 bp, and the number of encoded amino acids varied from 149 to 187. The derived molecular weights of the PbCaM proteins coded by the four genes ranged from 16.85 KDa to 21.20 KDa, and the isoelectric point values ranged from 3.88 to 4.31 (Table 1). Although there are several criteria for classifying CaM proteins [4, 7, 12], based on high degree of sequence similarity of the CaMs to known CaMs from different species [4–7, 12], the four pear CaM proteins are considered as canonical CaM proteins in pear (Table 1), which can well fit the trend of expansion of CaM in the green lineage ranged from 1 to 7 previously reported [12]. The sequence identity of PbCaM proteins versus AtCaM2 was within 88.6–99.3% (Table 1). Notably, each PbCaM protein contained four EF-hand domains and possessed similar secondary structure elements than* Arabidopsis* CaMs (Figure 1(a) and Figure S1).

### 3.2. Sequence Features and Organization of PbCaM Genes

Multiple sequence alignments of CaMs proteins from pear and* Arabidopsis* indicated that both pear and* Arabidopsis* CaMs contain highly similar distributions of conserved domains and motifs (Figure 1). The four conserved common helix-loop-helix structural motifs (the EF-hands), which act as Ca^2+^ binding sites of PbCaMs and AtCaMs, are illustrated in Figure 1(a). This architecture is consistent with the canonical “EF-hand” domain, which is composed of two alpha helices linked by a loop of 12 residues that usually binds Ca^2+^ [6, 7]. In an EF-hand loop, the calcium ion is coordinated in a pentagonal bipyramidal configuration through six residues in positions of 1, 3, 5, 7, 9, and 12 of loop. When an EF-hand motif binds to Ca^2+^, a conformational change of the EF-hand will be induced, leading to the activation or inactivation of target proteins [6, 7]. All of the pear and* Arabidopsis* CaM proteins possess four conserved motifs and similar distributions of motifs (Figure 1(b)), further supporting that the structural characteristics of CaMs in pear and* Arabidopsis* are highly conserved. Moreover, each CaM protein possesses four kinds of different motifs containing two motifs 1s, two motifs 2s, one motif 3, and one motif 4 (Figure 1(b)). The motif length ranged from 6 to 50, and the corresponding site number ranged from 12 to 24 (Table S2). Among the four motifs, motif 2, which is 50 amino acids in length, has been annotated as a CaM-binding domain with two EF-hands. The functional annotations of motifs 2, 3, and 4, however, have not been designated (Table S2). Although the functional annotation of motif 1 is unclear, its presence is closely adjacent to a CaM-binding domain (Figure 1(b)), suggesting that it may be related to recognition of calcium binding.

**Figure 2:**
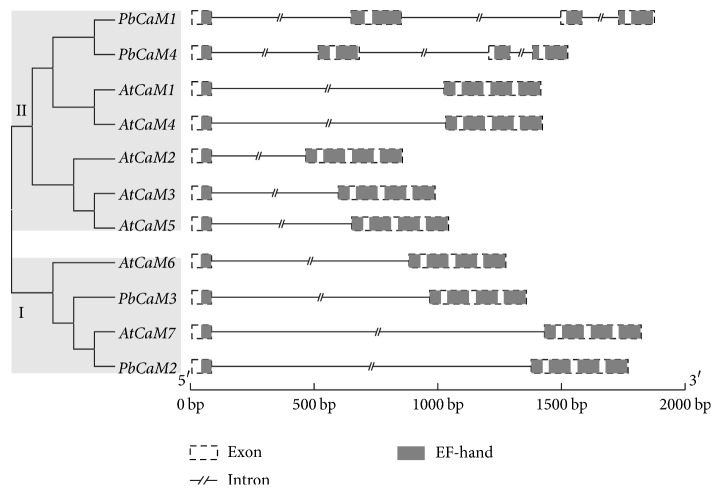
Organization and structure of* CaM* genes in pear (*P. bretschneideri*) and* A. thaliana*. Phylogenetic tree of the* PbCaM* family in pear was generated using the maximum-likelihood method and bootstrap analysis with 1000 replicates in MEGA 7.0. Gene structure was detected by GSDS 2.0 (http://gsds.cbi.pku.edu.cn/).

The full-length alignment of CaM family protein sequences in pear and* Arabidopsis* and their associated genomic and coding sequences were used to construct a ML phylogenetic tree and to examine gene structure. The 11 CaM proteins from pear and* Arabidopsis* can be divided into two major groups (I and II) on the basis of their phylogeny and gene structure (Figure 2). Phylogenetic analysis indicated that group I contains two PbCaM members (PbCaM2 and PbCaM3) and two AtCaM members (AtCaM6 and AtCaM7), while group II contains two PbCaM members (PbCaM1 and PbCaM4) and five AtCaM members (AtCaM1, AtCaM2, AtCaM3, AtCaM4, and AtCaM5), supported by the structures of CaM family members in each group. CaMs within the same group had a similar intron-exon distribution in* CaM* genes of both species. Most of the* CaM* genes (9/11) in* Arabidopsis* and pear contained two exons and one intron, with the exception of PbCaM1 and PbCaM4, which contained four exons and three introns. Group I genes contained one intron, two exons, and four EF-hands, and the intron was inserted in the first EF-hand (Figure 2), which is similar to AtCaM1–5 from group II, while in group II PbCaM1 and PbCaM4 contained three introns, four exons, and four EF-hands, respectively. Within group I, two introns were inserted in the architecture region of first EF-hand and third EF-hand, while AtCaM1–5 only contained one intron, two exons, and four EF-hands, and the intron was placed in the region of first EF-hand (Figure 2). However, the member from group I has a longer intron inserted than the ones from group II. Those data show that two subgroups of CaM proteins may possess different expansion patterns, group I only by changing size of inserting intron in the first EF-hand and group II by insertion of intron in the first EF-hand or the third EF-hand. These results revealed that the orthologous expansion of these subfamily members is highly restricted, especially in group I, which may possess more conserved functions. Interestingly, all* CaM* genes in pear were disrupted by the first intron at the Gly26 codon, which has been previously reported in* Arabidopsis* and solanaceous CaMs [7, 26]. The majority of the first intron is located in the region of the EF-hand 1, while the second exon encodes multiple EF-hands (Figure 2). Although the size of the introns in pear* CaM* genes is generally greater than those found within* Arabidopsis CaM* genes (Figure 2), the lengths of encoded proteins, however, are similar (Figure 1). Altogether the conserved exon/intron structure and motif distributions in* CaM* genes in pear and* Arabidopsis* testify the conserved expansion of CaM family members between pear and* Arabidopsis*.

### 3.3. Phylogenetic Analysis of CaM Orthologs during Several Plant Species

**Figure 3:**
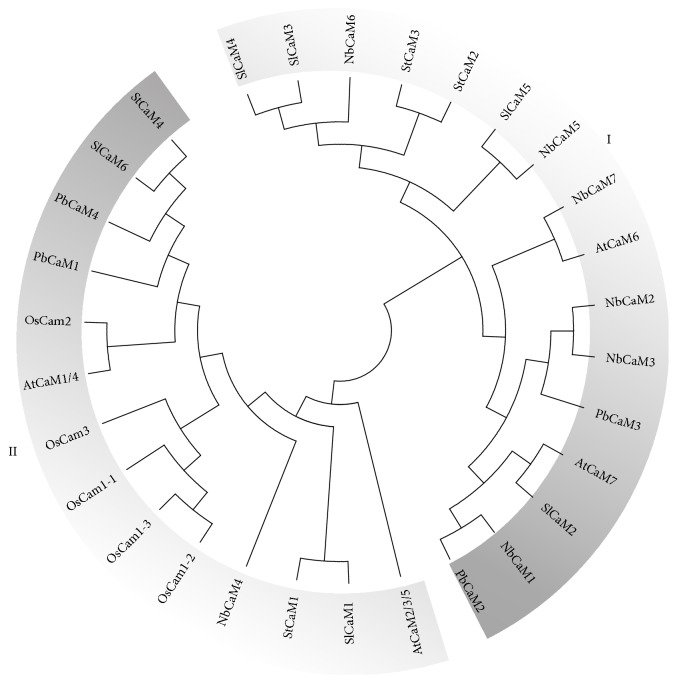
Phylogenetic analysis of the CaM protein family in six plant species. A phylogenetic tree of CaM proteins from six plants species (pear,* Arabidopsis*, rice, tomato, tobacco, and potato) was generated in ClustalW 2.0 using the maximum-likelihood method and bootstrap analysis with 1000 replicates in MEGA 7.0 based on the alignments of amino acids from full-length proteins. At,* Arabidopsis thaliana*; Nb,* Nicotiana benthamiana*; Os,* Oryza sativa*; Pb,* Pyrus bretschneideri*; Sl,* Solanum lycopersicum*; St,* Solanum tuberosum*.

To further examine the evolution of the CaM protein family, full-length amino acid sequences of pear,* Arabidopsis*, rice, tomato, potato, and tobacco CaMs that all contained four EF-hand domains, were aligned and then used to conduct a phylogenetic analysis. In a maximum-likelihood (ML) phylogenetic tree, the 30 CaM proteins included four CaMs from pear, four CaMs from* Arabidopsis* (seven* CaM* genes encode four proteins: AtCaM2, -3, -5 are homologous and AtCaM1 and AtCaM2 are homologous), five from rice, seven from tobacco, six from tomato, and four from potato. Collectively, the CaM proteins clustered into two major groups: I and II (Figure 3). The phylogenetic clusters of pear and* Arabidopsis* CaMs are consistent with the phylogenetic groups constructed from all six plant species. As shown in Figure 3, five of the six species contained two clusters, with rice being the exception. In the clustering analysis of the six species, group II included two of the four PbCaMs, two of the four AtCaMs, two of the six SlCaMs; two of the four StCaMs, and one of the seven NbCaMs. All five of the rice CaMs clustered in group II, while the Solanaceous CaMs clustered mainly in group I. Group I contained four of the six SlCaMs; two of the four StCaMs, and six of the seven NbCaMs, those members had more closely clustering blanches than the others from group I (Figure 3). The clustering analysis conducted in the present study supports a previous report that CaM proteins from Solanaceous species tend to cluster within a single phylogenetic clade [26]. It is notable that pear,* Arabidopsis*, and rice CaMs clustered differently. All of the pear and* Arabidopsis* CaMs clustered in either group I or II, while all of the rice CaMs were clustered exclusively in group II. These observations suggest that the CaM proteins in the six plant species evolved from two ancestral forms. It is plausible that the* CaM* genes from pear,* Arabidopsis*, tomato, potato, and tobacco that clustered into two groups may have evolved from the two ancestral forms of CaMs, whereas the species that cluster into just one of the phylogenetic groups may have been derived from either one of the two ancestral forms. A final determination of whether CaMs have evolved from two ancestral genes, however, still requires more supportive data.

### 3.4. Chromosome Locations and Synteny Analysis of PbCaM and AtCaM Genes

**Figure 4:**
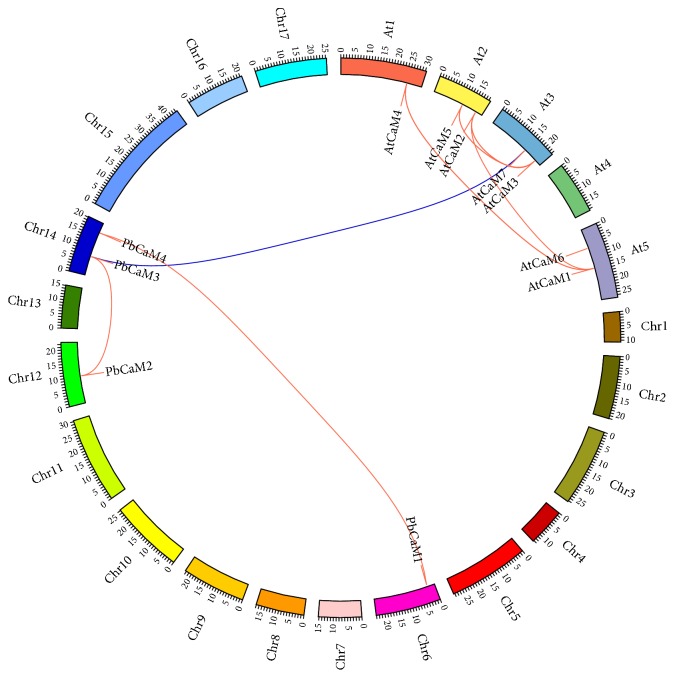
Synteny analysis of* CaM* gene family in pear (*P. bretschneideri*) and* A. thaliana*. The five* PbCaM* genes were located on four of the 17 pear chromosomes. The duplicated* CaM *gene pairs in pear and* Arabidopsis* are illustrated on pear chromosomes (Chr1–17) and* Arabidopsis* chromosomes (At1–5). Paralogous relationships of* PbCaM* and* AtCaM* genes are indicated by red solid color lines. The orthologous relationship of* CaM* genes between pear and* Arabidopsis* is indicated by blue solid color lines.

Chromosome localization analysis revealed that the four* PbCaM*s and seven* AtCaM*s mapped to four chromosomes in the pear and* Arabidopsis* genomes. Synteny analysis indicated seven paralogous duplications and one orthologous duplication within pear and* Arabidopsis*, and between pear and* Arabidopsis* (Table 2). The chromosome locations and synteny relationships of duplicated* CaM* gene pairs are shown in Figure 4 using Circos [37].* PbCaM3* and* PbCaM4* are located on chromosome 14, while the other two* PbCaM* genes (*PbCaM1 and PbCaM2*) mapped onto chromosomes 6 and 12, respectively (Figure 4 and Table 1). In comparison,* Arabidopsis* chromosomes 2, 3, and 5 possessed two* CaM* genes, respectively, while only* AtCaM4* was located on chromosome 1 (Figure 4). The* PbCaM* gene family contained two pairs of paralogous duplications (*PbCaM1*–*PbCaM4* and* PbCaM2*-*PbCaM3*), while the* AtCaM* gene family contained four pairs of paralogous duplications (*AtCaM1-AtCaM2*,* AtCaM1*–*AtCaM4*,* AtCaM2-AtCaM3*, and* AtCaM3*–*AtCaM5*). Only one orthologous duplication pair (*AtCaM7-PbCaM3*) was found between pear and* Arabidopsis* (Table 2). The different number of* CaM* gene duplication events in pear and* Arabidopsis* suggests that the* CaM* genes may undergo different duplications in two species. However, the presence of the orthologous duplication event between two species implies that the orthologous duplication or vertical evolution of* CaM* genes in two species has occurred during the long-term plant evolution processes. The paralogous and orthologous duplications of the* CaM*s within/between pear and* Arabidopsis* demonstrate the conserved relationships of* CaM* genes in pear and* Arabidopsis*, further suggesting that the* CaM* orthologs in pear may have similar or identical biological roles to mediate the transduction of calcium signals in plant cells.

### 3.5. Cis-Regulatory Element Analysis of PbCaM and AtCaM Genes

**Figure 5:**
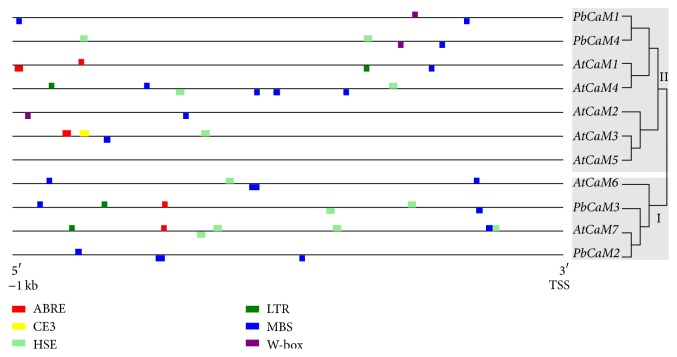
Stress-related cis-element analysis of the promoter regions of pear (*Pyrus bretschneideri*) and* Arabidopsis CaM* genes. Stress-related cis-elements including ABRE (involved in the response to ABA), CE3 (involved in ABA and VP1 responsiveness), HSE (involved in response to heat and oxidative stress), LTR (involved in response to low temperature), MBS (MYB binding site involved in response to ABA and drought), and W-box (WRKY binding site involved in abiotic stress and defense response) are shown in the promoter region (1000 bp upstream) of pear and* Arabidopsis CaM* genes. The colored boxes present above or below the line indicate the plus or minus strand for the distribution of* PbCaM* genes.

Stress-related cis-acting elements such as MBS (MYB transcription factor binding site), HSE (heat shock element), ABRE (ABA-responsive element), LTRE (low-temperature responsive element), CE3 (coupling element 3), and W-box (WRKY transcription factor binding site) have been demonstrated to play a key role in the response of plants to stresses [44–48]. The promoter regions (1000 bp upstream of the translation start site) of pear and* Arabidopsis CaM* genes were analyzed to exhibit distributions of conserved stress-related cis-elements on the promoter region of four of the* PbCaM* and seven of the* AtCaM* genes (Figure 5 and Table S3). Results indicated that the promoters of* CaM* genes from the same phylogenetic cluster contained similar distribution of stress-related cis-elements (Figure 5 and Table S3), which may be associated with the similar functions within the same group. Among the total of 44 occurrences of stress-related cis-elements, the ranked three were MBS (19), HSE (11), and ABRE (6) elements, followed by four LTR, three W-box, and one CE3, respectively (Figure 5 and Table S3). The number of stress-responsive elements in the promoter region of the four* PbCaMs* ranged from a maximum of six in* PbCaM3* to a minimum of three in* PbCaM1* and* PbCaM2*, while the numbers ranged from 2 to 7 in* Arabidopsis*, which indicated that CaM gene promoter regions possessed the similar components of cis-elements in pear and* Arabidopsis*. Interestingly, a total of 19 MBS elements were found in the promoter region of the pear and* Arabidopsis CaM* genes (Table S3) of eight MBS elements were present in four* PbCaMs* (one in* AtCaM1*,* AtCaM2*,* AtCaM3* or* AtCaM7*, four in* AtCaM4*, three in* AtCaM6*) and the remaining 11 MBS elements in six* AtCaM*s (two in* AtCaM1*, two in* AtCaM3*, and one in* AtCaM7*). This result suggests that the expression of* AtCaM*s and* PbCaMs* may be similarly induced by abiotic stresses. Moreover, only one CE3 element, involved in ABA signal transduction [44], was found in the promoter region of* AtCaM3* and none in* PbCaM* genes (Figure 5 and Table S2). These data suggest that the consistent or similar distribution of stress-related cis-elements in the promoter regions of both* PbCaM*s and* AtCaM*s may respond to similar environmental stimuli.

### 3.6. Expression Pattern of PbCaM Genes in Response to Salt and Osmotic Stress

**Figure 6:**
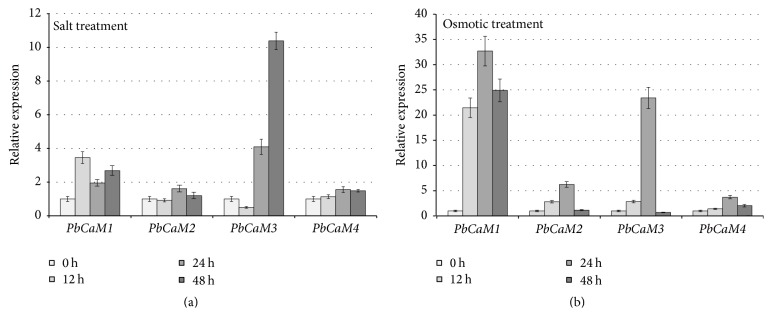
Expression of* PbCaM* genes in response to salt and osmotic stress. Shoots with young leaves were exposed to 1/2 MS solution containing 200 mM NaCl or 15% (w/v) polyethylene glycol (PEG6000) for the salt treatment (a) or osmotic treatment (b). Leaves were sampled at 0, 12, 24, and 48 h and were subsequently used for QRT-qCR analysis. All of the experiments were examined using three biological replicates and three technical replicates for all of the genes. The data was shown using the mean ± standard deviation (SD). The error bars represent the SDs of nine replicates.

The temporal expression pattern of genes or gene families has been widely used to identify candidate genes in response to salt stress and osmotic stress [38, 49–51]. The qRT-PCR data indicated that the expression profiles of* PbCaM* genes varied over time (0, 12, 24, and 48 h) in response to salt and osmotic stress (Figure 6). All four* PbCaM* genes were upregulated with different degrees in response to salt and osmotic stresses. Two* PbCaM* genes (*PbCaM1* and* PbCaM4*) were significantly upregulated in response to salt or osmotic stress (Figure 6). Moreover,* PbCaM1*,* PbCaM3,* and* PbCaM4* exhibited similar upregulatory patterns from 0 h to 48 h after being exposed to the salt or osmotic stress, while* PbCaM2* was induced with different degrees of upregulation during salt and osmotic from 0 h to 48 (Figure 6(a)), which may imply the subfunctionalization of CaM genes in pear.

**Figure 7:**
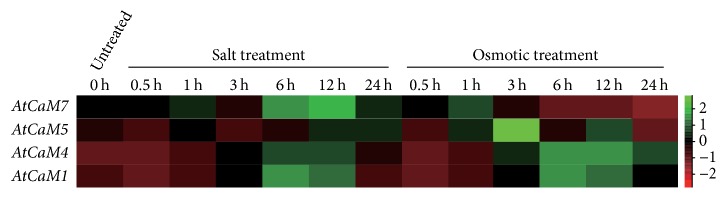
Expression of* AtCaM* genes in response to salt and osmotic stress. Expression data of* CaM* genes in* Arabidopsis* collected from the AtGenExpress (http://jsp.weigelworld.org/AtGenExpress/resources/) and heat map generated using Cluster 3.0 software. Bar on the left represents log2 transformed values, ranging from −2 to 2, represent from low to high expression.

All four* PbCaM* genes were differentially upregulated in response to salt and osmotic treatments from 0 h to 48 h. Although two of four genes (*PbCaM1* and* PbCaM3*) were consistently upregulated by both salt and osmotic stresses, they showed higher inductive expression under osmotic stress treatment than under salt stress treatment (Figure 6), suggesting that these two* PbCaM* genes may be more sensitive to osmotic stress than to salt stress. The similar cis-element distributions in the* PbCaM* promoters further support this inference (Figure 5). Therefore, it is reasonable to consider that both* PbCaM1* and* PbCaM3* may play critical roles in the response to osmotic and/or salt stress. This is further supported by the fact that the expressions of* PbCaM* orthologs in* Arabidopsis*, based on data available from AtGenExpress (http://jsp.weigelworld.org/AtGenExpress/resources/) [42],* AtCaM1* (*PbCaM1* ortholog), and* AtCaM7* (*PbCaM3* ortholog) were also obviously induced from 0 to 24 h during salt and osmotic stress (Figure 7 and Table S4), further suggesting that* PbCaM1* and* PbCaM3* have evolved similar role or subfunctionalization in response to salt and osmotic stress in pear. In Figure 7, the other five genes in* Arabidopsis* were induced and expressed under salt and osmotic stresses, indicating that the most* AtCaM* genes exhibited similar or differential expression trends after being exposed to the same treatments in different treatment times. These data further indicated that CaM genes play conserved roles in response to abiotic stresses in plants.

## 4. Conclusions

Through comprehensive analysis of* CaM* genes in pear, we identified and cloned and four putative canonical CaM genes in pear. We further performed a genome-wide analysis of gene structure, gene duplication, synteny, and stress-responsive expression for putative PbCaM genes in comparison to Arabidopsis CaM genes to elucidate the possible expansion patterns of pear CaM genes in pear. Phylogenetic analysis of* CaM* genes from several sequenced species indicated a conserved evolution of the* CaM* gene family. Results also indicated that pear* CaM* genes were more closely related to* Arabidopsis CaM* genes then they were to* CaM* genes in other plant species.* PbCaM* and* AtCaM* had a similar distribution of cis-elements and expressions in responses to salt and osmotic stress. All four PbCaMs had been differentially upregulated expression under salt stress and osmotic stress. In particular,* PbCaM1* and* PbCaM3* were both significantly upregulated in response to salt and osmotic stress, suggesting they may play an important role in the common response of pear to these stresses. The present study provides basic information on the composition, structure, and expression of pear* CaM* genes in response to salt and osmotic stresses, which can be used as a foundation for future studies regarding the specific function of* CaM* genes in pear.

## Supplementary Material

Table S1. Primers for RT-PCR cloning and qRT-PCR. Table S2. Motif analysis of CaM family members. Table S3. Stress-responsive cis-element analysis of the promoter region of CaM family genes in pear and Arabidopsis. Table S4. Expression of AtCaM genes in response to salt and osmotic stresses. Figure S1. Secondary structure comparison of PbCaMs and AtCaMs.

## Figures and Tables

**Table 1:** Characteristics of *CaM* genes in pear (*P. bretschneideri*).

Gene name	Gene ID	Accession ID	Position	CDS (bP)	Size (aa)	Number of introns	MW (KDa)	PI	Number of EF-hands	% identity to AtCaM2
*PbCaM1*	Pbr028327.2	KU950328	Chr6: 2648292…2650176 (+)	564	187	4	21.20	4.31	4	88.62
*PbCaM2*	Pbr039046.1	KU950329	Chr12: 10152516…10154840 (−)	450	149	3	16.85	3.88	4	99.33
*PbCaM3*	Pbr038169.1	KU950330	Chr14: 7176273…7178095 (−)	450	149	1	16.85	3.88	4	99.33
*PbCaM4*	Pbr038805.1	KU950331	Chr14: 16928793…16930245 (+)	450	149	3	16.97	3.90	4	87.92

**Table 2:** Gene duplication and synteny analysis of *CaM* genes among *P. bretschneideri* and *A. thaliana*.

Duplicated gene 1	Duplicated gene 2	Synteny relationship
*AtCaM1* (AT5G37780)	*AtCaM2* (AT2G41110)	Paralogous
*AtCaM2* (AT2G41110)	*AtCaM3* (AT3G56800)	Paralogous
*AtCaM3* (AT3G56800)	*AtCaM2* (AT2G41110)	Paralogous
*AtCaM3* (AT3G56800)	*AtCaM5* (AT2G27030)	Paralogous
*AtCaM5 *(AT2G27030)	*AtCaM3* (AT3G56800)	Paralogous
*PbCaM1* (Pbr012900.1)	*PbCaM5* (Pbr018864.1)	Paralogous
*PbCaM2* (Pbr039046.1)	*PbCaM3* (Pbr038169.1)	Paralogous
*AtCaM7* (AT3G43810)	*PbCaM3* (Pbr038169.1)	Orthologous
